# Effect of Storage Conditions on the Long-Term Stability of Bactericidal Effects for Laser Generated Silver Nanoparticles

**DOI:** 10.3390/nano8040218

**Published:** 2018-04-04

**Authors:** Peri Korshed, Lin Li, Duc-The Ngo, Tao Wang

**Affiliations:** 1 School of Biological Sciences, Faculty of Biology, Medicine and Health, The University of Manchester, Oxford Road, Manchester M13 9PT, UK; peri.korshed@postgrad.manchester.ac.uk; 2 Laser Processing Research Centre, School of Mechanical, Aerospace and Civil Engineering, The University of Manchester, Manchester M13 9PL, UK; lin.li@manchester.ac.uk; 3 Electron Microscopy Centre, School of Materials, University of Manchester, Manchester M13 9PL, UK; duc-the.ngo@manchester.ac.uk

**Keywords:** silver nanoparticles, antibacterial durability, nanoparticle stability, *E. coli*, nanoparticle storage, laser nanoparticles

## Abstract

Silver nanoparticles (AgNPs) are widely used as antibacterial agents, but their antibacterial durability and the influence by storage conditions have not been thoroughly investigated. In this study, AgNPs were produced using a picosecond laser and stored under three different conditions: daylight, dark and cold (4 °C). The antibacterial effects of the laser AgNPs were examined against *Escherichia coli* in either a 14-day interval (frequent air exposure) or a 45-day interval (less frequent air exposure) using a well-diffusion method until the antibacterial effects disappeared. Results showed that the antibacterial activity of the laser generated AgNPs lasted 266 to 405 days. Frequent air exposure increased particle oxidation as measured by high-angle annular dark-field detector for scanning transmission electron microscopy (HAADF-STEM) and X-ray energy dispersive (EDX) spectroscopy, and reduced the antibacterial duration by about 13 weeks. Compared to the chemically produced AgNPs, the antibacterial effect of the laser AgNPs lasted over 100 days longer when tested in the 45-day interval, but was susceptible to oxidation when frequently exposed to the air. The laser generated AgNPs had lower antibacterial activity when stored in cold compared to that stored at room temperature. This study demonstrated the long lasting antibacterial durability of the laser generated AgNPs. Such information could help design future medical applications for the AgNPs.

## 1. Introduction

Silver nanoparticles (AgNPs) are well known antibacterial agents that function against a wide spectrum of Gram-positive and Gram-negative bacterial strains [1,2,3]. However, the lack of stability of AgNPs has prevented the material from wider medical or hygienic applications [3,4,5]. A number of attempts were made to evaluate the stability of AgNPs [6,7,8,9], but there has not been a general conclusion on the antibacterial duration for AgNPs. To maximise the application potential of AgNPs, it is necessary to understand the shelf life of the material under different storage conditions.

Studies addressing the durability of AgNPs have largely focused on the duration of AgNPs that had been immobilised on the supporting materials [10]. For example, antibacterial textiles were produced by incorporating AgNPs into cotton fabrics. It was found that the AgNP-embedded cotton fabrics could withstand 30–50 sequent laundering cycles without losing their antibacterial effect against *S. aureus* and *E. coli* [11].

Efforts have also been made to determine changes of the physical properties of the AgNPs during or after storage [12,13]. It was reported that the physiochemical properties of AgNPs, such as agglomeration, zeta potential and Ag ion (Ag^+^) release, were differentially altered during a six-month storage period, which contributed to the “aging” effect of the AgNPs, influencing mammalian cell toxicity [14]. Oxidative dissolution of AgNPs closely correlates to the durability of AgNPs. AgNPs are sensitive to oxygen which leads to partial dissolution of AgNPs, releasing Ag^+^ [15,16,17]. The amount of Ag^+^ release has a time-dependent increase [18] due to slow dissolution of Ag ion during storage [17]. The agglomeration and Ag^+^ release likely influences the morphology and size of AgNPs which were observed after a long term (100 days) storage of AgNPs [19].

To prolong the duration of the functionality, AgNPs have also been embedded into other materials, such as polymers. For example, AgNPs were immobilised between the polydopamine (PDA) bilayers that were coated on the silicon urinary catheter surfaces, which has significantly reduced colonisations of uropathogens [20]. However, data from this type of study did not provide sufficient information on the antibacterial durability of the raw AgNPs.

Although the durability of AgNPs was addressed from different angles in the literature, very few studies were designed to directly and systemically evaluate the impact of the length of storage and storage conditions on the antibacterial activity of AgNPs. It is likely that manufacturing methods could also influence the stability of the AgNPs.

We have recently produced AgNPs using different types of laser ablation techniques, and conducted a series of studies on the antibacterial activities of the laser AgNPs and the associated mechanisms [21,22]. However, the duration of the antibacterial effects for the laser generated AgNPs has not been thoroughly evaluated and compared to the AgNPs made by the conventional chemical method. Here we report a study where the laser generated AgNPs were stored under three different conditions: daylight at room temperature, dark at room temperature, and cold condition in a 4 °C fridge. The antibacterial effects were determined in two regular intervals, i.e., every 14 days or every 45 days, respectively, until the effects completely disappeared. The two different testing intervals have also simultaneously created different frequencies of air exposure to the samples. Results showed that the bactericidal effect of laser generated AgNPs could last for over a year. The antibacterial duration was more significantly influenced by air exposure of the NPs than by other storage conditions such as light and temperature, which correlated to the surface oxidation of the NPs. The antibacterial durability of all conditions was also compared to that of commercially purchased AgNPs made from chemical methods. Information obtained from the study would contribute to future design of biomedical applications that involves AgNPs.

## 2. Materials and Methods

### 2.1. Nanoparticles Production

Nanoparticle production by pulsed picosecond laser ablation in deionised water (dH_2_O) was described in our previous publication [21]. Briefly, Ag plates (dimensions of 25 mm × 25 mm × 2 mm, purity 99.99%) were sterilised by immersion into ethanol and then autoclaved with dH_2_O. The Ag plates were then placed into a glass vessel containing 20 mL of dH_2_O at a level of 2 mm above them. A picosecond pulsed Nd: YVO4 laser with a wavelength of 1064 nm was used to ablate the plate at a pulse repetition rate of 200 kHz and an average power of 9.12 W. The average size for AgNPs produced was 27.2 nm, ranging from 10–70 nm [21].

The chemically produced AgNPs (concentration 20 μg/mL and average size about 35 nm) were purchased from Sigma-Aldrich (Dorset, UK).

### 2.2. Bacteria Culture and the Determination of the Antibacterial Activities of NPs

Bacterial strain *E. coli* (JM 109) [22] was purchased from Promega (Southampton, UK). A single colony of bacterial cells was inoculated in 10 mL of autoclaved Muller–Hinton broth media (Sigma-Aldrich, Dorset, UK), and incubated at 37 °C overnight with shaking at 225 rpm. The bacteria suspension was diluted to give 10^4^ cfu/mL ready to be used for the antibacterial experiments described below. The antibacterial activities of NPs were determined following the standard Nathan’s Agar Well Diffusion (NAWD) technique. Briefly, a lawn of bacterial culture prepared above was spread uniformly on the Muller–Hinton agar plates using sterile cotton swabs and left for 10 min for culture absorption. Multiple 6 mm wells were created by punching the bacteria coated Muller–Hinton agar plates using a cylinder glass tube. Fifty microliters of NP sample solution was added into each well and was incubated at 37 °C for 18 h. The zones of inhibition (ZOI), which reflects the susceptibility of microbes to the NPs, were then measured [23].

### 2.3. Storage Conditions

The same batch of AgNPs prepared was divided into 6 samples to be used for testing the impact of different storage conditions and the frequencies of air exposure on the antibacterial effects (Table 1). Three storage conditions were used: (1) daylight at room temperature, (2) dark (wrapped by foil) at room temperature, and (3) cold condition (stored in a 4 °C fridge). Under each storage condition, the antibacterial effect was tested either every 14 days where the sample was frequently opened and exposed to the air for testing, or every 45 days where the sample were relatively less frequently exposed to the air.

### 2.4. NP’s Morphology and Elemental Composition

Morphology and elemental composition of the Ag nanoparticles were characterised using a FEI Tecnai F30 transmission electron microscope (TEM) with a field emission gun (FEG) operating at a 300 kV accelerated voltage. The microscope was equipped with a Fischione high-angle annular dark-field (HAADF) detector for scanning transmission electron microscopy (STEM) imaging, and an X-Max 80 T (Oxford Instruments) silicon drift detector (SDD) for X-ray energy dispersive (EDX) spectroscopy. Samples for TEM characterization were prepared by placing a drop of nanoparticles colloidal onto a copper grid supported with a holey carbon film, then naturally dried at room temperature.

### 2.5. Statistical Analysis

Data in this study was presented as mean ± SEM. One-way ANOVA followed by Tukey’s Post-hoc test was conducted for all data to determine the significance of differences between the samples. *p* ≤ 0.05 was considered as statistically significant. Each experiment was performed in triplicate.

## 3. Results

### 3.1. Duration of the Antibacterial Effects of Laser Generated AgNPs

**Figure 1:**
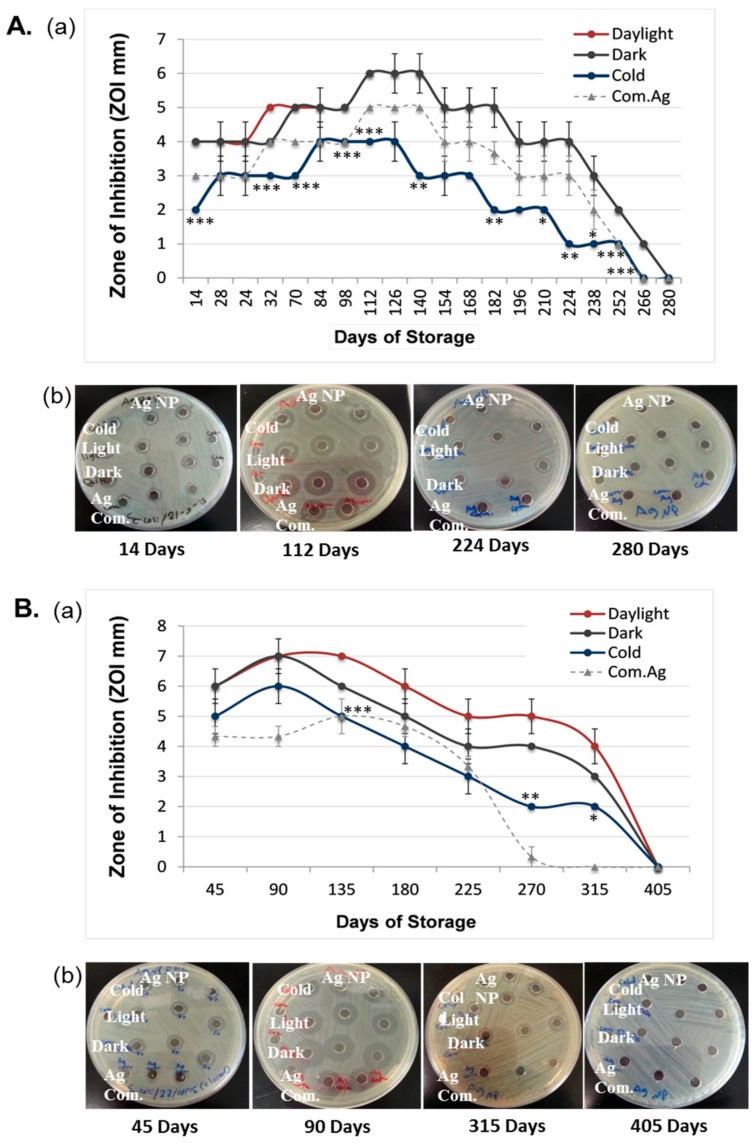
Antibacterial duration of laser generated silver nanoparticles (AgNPs). Samples of laser generated AgNPs were stored under three different conditions: light, dark, and cold. The antibacterial activities of the NPs were measured using the well-diffusion method every 14 days (A, the Open-14 samples) or every 45 days (B, the Open-45 samples). A thin layer of *E. coli* bacterial cells were grown on Muller–Hinton agar plates and then six-millimeter wells were created through the agar. Fifty microliters of laser AgNPs concentration (50 µg/mL) were added to each well in triplicate. The plates were incubated at 37 °C for 24 h. Zones of inhibition (ZOI) were measured (**Ab** and **Bb**) and plotted against days of storage (**Aa** and **Ba**). The antibacterial effects of chemically produced AgNPs that were purchased from a commercial source (Com.Ag) and stored in cold were also measured in parallel. Data are presented as mean ± SE. Compared to the samples that were measured on the same day but stored under different conditions, * *p* ≤ 0.05, ** *p* ≤ 0.01, *** *p* ≤ 0.001, *n* = 3.

The duration of the antibacterial effect of laser generated AgNPs were tested against *E. coli*. Results showed that the laser generated AgNPs under different storage conditions lost their antibacterial effects around one year (between 266 days and 405 days, Figure 1). Samples that were stored at daylight and dark at room temperature had similar antibacterial effects (Figure 1). However, samples stored in cold (4 °C) had consistently smaller ZOI at most of testing time points (Figure 1), especially for samples that were tested every 14 days (Figure 1A).

### 3.2. Frequent Air Exposure on the Antibacterial Effects of Laser Generated AgNPs

**Figure 2:**
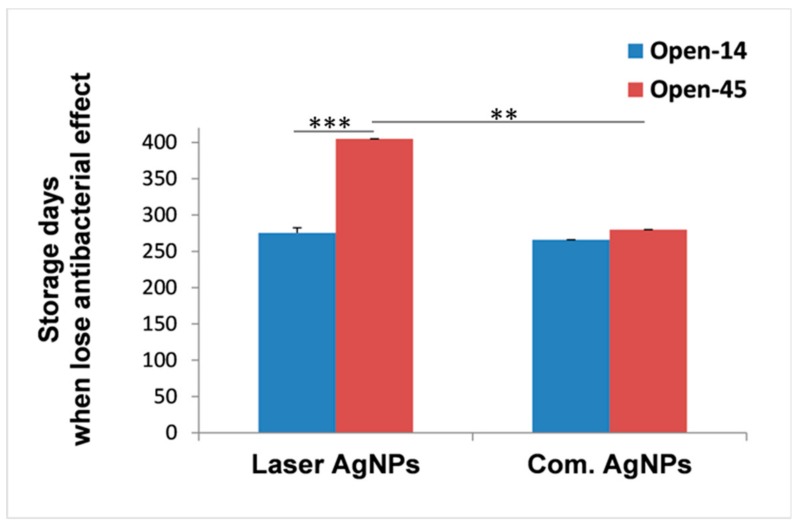
Impact of air exposure on the duration of antibacterial activities of AgNPs. The antibacterial effects of laser generated AgNPs or chemically generated AgNPs (Com.AgNPs) were determined every 14 days (Open-14) or every 45 days (Open-45) using the well-diffusion method until the effects completely disappeared. The average numbers of days taken for the AgNPs to loss their antibacterial activities were compared between the Open-14 and Open-45 samples regardless the storage conditions. Data are presented as mean ± SE, ** *p* ≤ 0.01, *** *p* ≤ 0.001, *n* = 3.

To determine whether more frequent air exposure could change the duration of the antibacterial effects, antibacterial activities of the NPs under the three storage conditions were tested in either a 14-day interval (Open-14) or a 45-day interval (Open-45). Sampling every 14 days required more frequent opening of the sample vials than sampling every 45 days, thus, giving more frequent air exposure of the samples. Results revealed that the antibacterial effect of the Open-14 samples stopped much earlier as compared to the Open-45 samples (Figure 2). The Open-14 samples took an average of 275.3 days (between 260 and 280 days) to completely lose their bactericidal effects (Figure 2), which was nearly five months earlier than the time required for the Open-45 samples (405 days), regardless storage conditions (Figure 1Aa,Ba and Figure 2).

**Figure 3:**
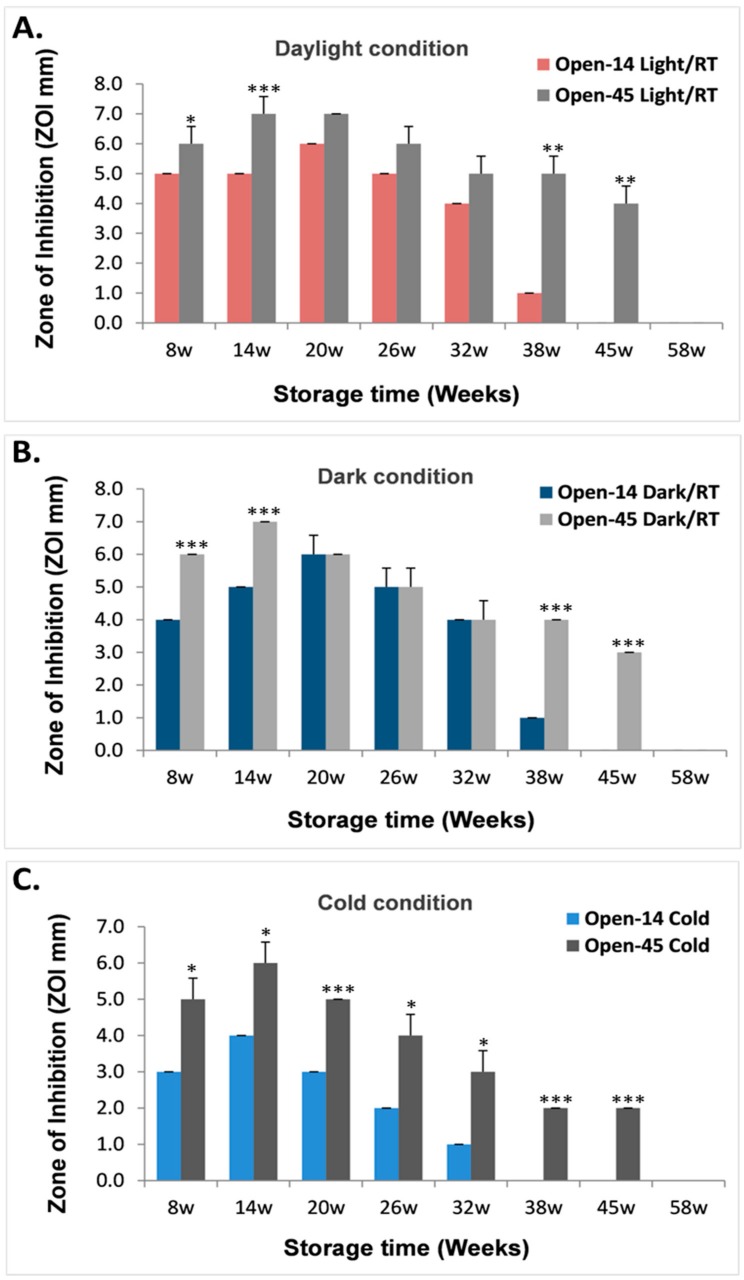
The effect of frequent air exposure of laser generated AgNPs on its antibacterial activities under different storage conditions. The laser generated AgNPs were stored under three different conditions: daylight (**A**) and dark (**B**) at room temperature (RT) and cold at 4 °C (**C**). The antibacterial effects of AgNPs were determined every 14 days (Open-14) or every 45 days (Open-45) using the well-diffusion method, and results were presented when the tests were conducted in the same week. Data are mean ± SE. * *p* ≤ 0.05, ** *p* ≤ 0.01, *** *p* ≤ 0.001, *n* = 3.

To further understand the impact of air exposure on the antibacterial durability for the laser generated AgNPs, we directly compared the antibacterial effects under each of the three different storage conditions between the Open-14 and Open-45 samples when the test happened in the same week of storage for the two samples (Figure 3).

For the AgNPs that were stored at room temperature under either daylight or dark conditions, the Open 45-samples (Open-45_Light/RT_ and Open-45_Dark/RT_) had either an equivalent or higher antibacterial effect compared to the Open-14 samples (Open-14_Light/RT_ and Open-14_Dark/RT_) during the course of storage (Figure 3A,B). However, for the laser AgNPs that were stored under cold condition, the Open-45_Cold_ samples had a significant higher antibacterial ability at each time point compared to the Open-14_Cold_ samples during the same course of storage (Figure 3C).

When stored at room temperature, the Open-14_Light/RT_ and Open-14_Dark/RT_ samples completely lost their antibacterial effects on week 45, while the antibacterial effects for the Open-45_Light/RT_ and Open-45_Dark/RT_ samples were able to continue until week 58, which was 13 weeks longer than that of the Open-14_RT_ samples (Figure 3A,B). This suggests that avoiding frequent air exposure could significantly prolong the antibacterial effect of the laser AgNPs, and this effect seemed independent of whether the sample had been stored under daylight or dark conditions (Figure 3A,B).

When stored under cold condition, the Open-45_Cold_ samples had similar length of antibacterial duration as the sample stored at room temperature (Open-45_Light/RT_ and Open-45_Dark/RT_), but the Open-14_Cold_ samples lost their antibacterial effects on week 38 (Figure 3C), which was much earlier than their counterparts stored at room temperature (45 weeks as described above, Figure 3A,B). These results suggest that the laser generated AgNPs were more susceptible to air exposure for maintaining their antibacterial property when stored in the cold.

### 3.3. Comparison of the Antibacterial Duration Between Laser and Chemically Generated AgNPs

We also measured the antibacterial duration for the commercially purchased AgNPs that were chemically produced and stored in sodium citrate and cold at 4 °C as recommended by the manufacture. Data were compared with what obtained from the laser generated AgNPs that were stored under the same temperature but in dH_2_O. We found that, when more frequently exposed to the air, the antibacterial durability for the commercial AgNPs (Open-14_Com.Ag_) was similar to the laser AgNPs (Figure 1A). The antibacterial effect for both types of AgNPs ended at the same testing time point (day 266) (Figure 1A). However, when the samples were less frequently exposed to the air (Open-45_Com.Ag_), the antibacterial effect of the commercial AgNPs ended between 270 and 315 days, which was earlier than that of the laser AgNPs (405 days, Figure 2). This result suggested that the laser generated NPs benefited more from preventing frequent air exposure.

**Figure 4:**
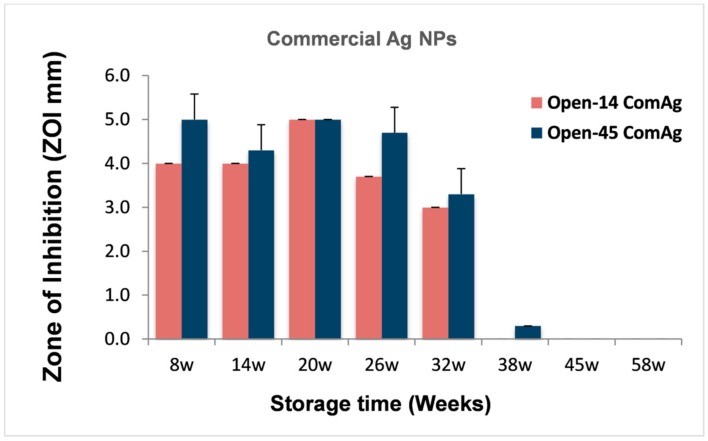
The effect of frequent air exposure on the antibacterial effect of the chemically produced AgNPs. The antibacterial effects of the chemically produced AgNPs that were purchased from a commercial source (Com.Ag) were determined every 14 days (Open-14) or every 45 days (Open-45) using the well-diffusion method, and results were presented when the measurement were conducted in the same week of storage. Data are mean ± SE, *n* = 3.

Additionally, we directly compared the antibacterial effects of chemically produced AgNPs between the Open-14_ComAg_ and Open-45_ComAg_ samples when the test happened in same week of storage (Figure 4). Frequent air exposure did not seem to have significant impact on the antibacterial effect of the commercial AgNPs, suggesting the commercial samples were reasonably stable.

### 3.4. Frequent Air Exposure Increase Oxidation of Laser Generated AgNPs

**Figure 5:**
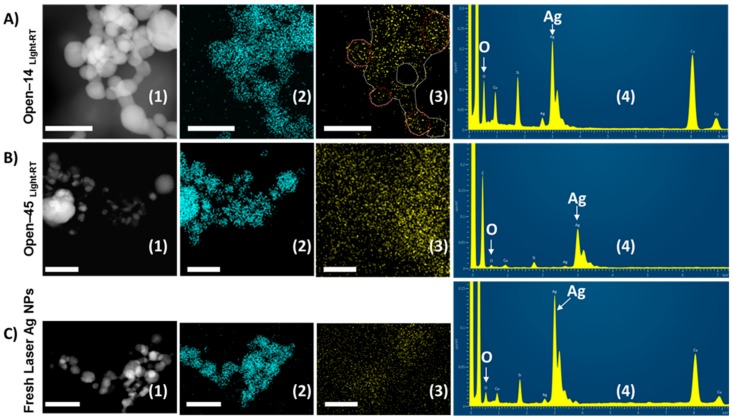
Elemental maps using scanning transmission electron microscopy (STEM)-X-ray energy dispersive (EDX) spectroscopy analysis for AgNPs. The laser generated AgNPs were stored under daylight condition at room temperature but subjected to the air exposure every 14 days (the Open-14_Light/RT_ sample, (**A1**–**A4**), or every 45 days (the Open-45_Light/RT_ sample, (**B1**–**B4**), in which: (**A1**–**C1**) STEM-high-angle annular dark-field detector (HAADF) images showing AgNPs, (**A2**–**C2**) Ag maps obtained from HAADF regions, (A**3**–**C3**) O maps obtained from HAADF regions; and (**A4**–**C4**) EDX spectra integrated from HAADF image regions. Fresh prepared laser AgNPs were presented in (**C1**–**C4**). All scale bars are 100 nm. The trace line on (**A3**) was manually produced to highlight the shape of oxygen map.

To explore the mechanisms behind the earlier loss of antibacterial properties when AgNPs were frequently exposed to the air, we conducted EDX spectroscopy on STEM to determine changes of the chemical composition on the nanoparticle surfaces after storage. To do so, an electron probe was scanned on the sample in a raster. In parallel with the STEM-HAADF image formed by collecting the transmitted beam, collection of EDX spectra from each pixel, where the e-beam scanned on, allows elemental maps to be constructed as shown in Figure 5. Figure 5 illustrates STEM-EDX elemental maps of O and Ag for the Open-14 and Open-45 laser AgNPs samples that had been stored at room temperature under daylight. Results were compared with the freshly prepared laser AgNPs.

The AgNPs were clearly seen in STEM-HAADF images (Figure 5A1–C1) whilst locations of detected elements, namely, Ag and O, are also visible from STEM-EDX maps (Figure 5A2–C2 and A3–C3). It was revealed that a well-defined map of oxygen was observed for the Open-14 samples (Figure 5A3), traced lines suggesting an apparent oxidation on the surface of the AgNPs. In contrast, noisy maps of oxygen were displayed for the Open-45 and fresh laser AgNPs samples (Figure 5B3,C3) which indicates an unlikely significant oxidation on the nanoparticles in those two samples. In this case, oxidation on the TEM specimens of those two samples (Figure 5B3,C3) was possible but the signals of oxygen from EDX spectra of those specimens were not high enough to construct a clear oxygen map compared to the highly defined shape of the O map for the Open-14 laser AgNPs in Figure 5A3.

Figure 5A4–C4 shows EDX spectra of the fresh AgNPs (Figure 5C4) and the AgNPs after being more (Open-14 sample, 5A4) or less frequently (Open-45 sample, Figure 5B4) exposed to the air at room temperature under daylight condition. Both O and Ag elements could be detected from the spectra of all samples. Additional elements appear on the spectra but were not from the samples themselves. For example, Cu comes from TEM grid used for sample preparations, and Si belongs to the glass ware used for storing and preparation of NPs under laser ablation. It could be seen from the spectra that intensity of the oxygen peak in the Open-14 sample (Figure 5A4) was significantly higher than that of both the Open-45 sample (Figure 5B4) and the fresh sample (Figure 5C4), suggesting an increased oxidation occurred in the Open-14 samples when more frequently been opened. This further supported the increased oxidation occurring on the AgNP surfaces when the samples were more frequently air exposed. The result was also in line with the shorter antibacterial period of the Open-14 sample compared to the Open-45 sample tested in the same condition (Figure 1).

## 4. Discussion

In this study, we demonstrated the role of storage conditions (daylight, dark, and cold) and frequency of air exposure under the three storage conditions in the antibacterial stability of laser generated AgNPs. This is the first study to evaluate the long-term antibacterial durability for the laser generated AgNPs. Results demonstrated that the antibacterial effect of AgNPs could last from 266 to 405 days depending on the frequency of air exposure and storage conditions. Samples that were stored in cold condition had consistently lower antibacterial activities than samples stored at room temperature. Frequent air exposure significantly reduced the antibacterial duration of the laser generated AgNPs by about five months. We have also detected increased oxidation of the AgNPs when frequently exposed to the air, which could contribute to the early loss of antibacterial ability.

It is known that Ag^+^ is a key component contributing to the antibacterial activity of AgNPs. Ag^+^ have strong affinity in binding to cellular components including proteins, sulfhydryl groups of essential metabolic enzymes, nucleic acids, and cell wall components. This leads to disruption of cell proliferation, membrane permeability and several other metabolic pathways within cells [24]. Ag^+^ release from NPs is likely influenced by temperature. It is plausible that a lower temperature could slow down the release of Ag^+^, which likely contributes to the relatively lower antibacterial activity of the laser AgNPs when being stored in the cold condition in our study.

The well-diffusion method is ideal for measuring the antibacterial effect of Ag^+^ that released from AgNPs. However, due to the limited mobility of NPs in agar, the well-diffusion measurement could underestimate the bactericidal effect of NPs via direct interaction with bacterial cells. This should be taken into account when interpreting the data.

Oxidative dissolution of silver nanoparticles is characteristic of AgNPs. This phenomenon could be observed by the colour change of the silver nanoparticle suspension [25]. The availability of oxygen is considered to be the main factor that affects Ag^+^ release [10,26,27]. Our results showed that the laser generated AgNPs, when frequently exposed to the air, had earlier termination of the antibacterial activities than those less frequently exposed to the air (Figure 3). This may be due to early depletion of Ag^+^ caused by accelerated dissolution of AgNPs by oxygen when frequently exposed to the air, shortening the antibacterial shelf life of the AgNPs. The EDX spectroscopy and STEM-EDX revealed obvious oxygen signal on the Open-14 samples, which strongly suggest the increased oxidation process on the surfaces of the AgNPs that were exposed frequently to the air. The oxidation of AgNPs also generates hydrogen peroxide (H_2_O_2_), which mediates the toxicity of AgNPs [28]. However, it is possible that laser ablation could also generate an Ag_2_O shell on the surface of some AgNPs. Future work could confirm the existence of such Ag_2_O in the laser produced AgNP population and determine their role in the antibacterial durability of the laser produced AgNPs.

We found that the antibacterial activity of laser generated AgNPs were similar when being stored under daylight and dark at room temperature. This contradicts some publications where AgNPs were described as photosensitive and susceptible to oxidation by daylight leading to AgNPs dissolution [29,30,31,32,33]. The study by Yu et al. suggested that exposure of AgNP to the sunlight leads to oxidation and release of Ag^+^ and formation of new AgNPs [33]. Another study by George et al. reported that exposure of AgNPs to daylight for up to 8 days caused surface oxidation and dissolution of AgNPs, while exposure of the same NPs to UV light leads to a decrease in AgNPs dissolution. Thus, exposure of AgNPs to the light (visible light or UV light) will lead to either oxidation or reduction of the AgNPs [31]. Although light could cause AgNP dissolution, oxidation is still the main factor contributing to AgNP dissolution. Light induced AgNP dissolution could readily be detected using various material characterisation tools, but its contribution to the overall antibacterial effect of the AgNP sample is likely minimum. In addition, the intensity of the light exposure, for example, the lighting condition in the laboratory and the geographical sunshine period, should be taken into account when interpreting data. In future work, controlled light conditions with different intensities could be employed to understand the influence of light to the antibacterial durability of AgNPs.

We observed that frequent air exposure did not significantly affect the antibacterial activity and durability of the commercial AgNPs (Figure 2) compared to the laser generated AgNPs. The commercial AgNPs were stored in sodium citrate that is a capping agent during the NP synthesis to control particle size and prevent agglomeration. Sodium citrate also has a weak buffering role to minimise pH change, which may protect the AgNPs from oxidative dissolution, thus accounting for the observed reduced sensitivity to air exposure. On the other hand, the laser AgNPs were produced in deionised water which has no reducing effect on the NPs [13]. However, this did not seem to have affected the antibacterial durability. Being produced and stored in clean water is an obvious advantage of the laser generated AgNPs. The laser method could avoid contaminations by agents that were carried over from the process of chemical synthesis, benefiting downstream medical applications. The presence of some ligands in water such as Cl^−^, and SO_4_^2−^ could affect the bioavailability of Ag^+^ by interaction with Ag^+^ causing its precipitation which makes the Ag^+^ less toxic, but these ligands could be very low in the fresh water or absent in deionised water [34].

## 5. Conclusions

In conclusion, the antibacterial activity of laser generated AgNPs lasted 266 days to 405 days depending on the degree of air exposure and storage conditions. Frequent air exposure increased particle oxidation and reduced the antibacterial durability of the laser generated AgNPs by about 13 weeks. When tested in a 45-day interval, the antibacterial effect of the laser AgNPs lasted over 100 days longer than the chemically produced AgNPs that were purchased from the commercial source. However, the laser AgNPs were susceptible to oxidation when frequently exposed to the air. The antibacterial results generated in this study were based on the ZOI from the well-diffusion method, which provided more sensitive measurements of the effect of Ag+ that were released from AgNPs.

## Figures and Tables

**Table 1:** Sample storage conditions and identities.

Sample ID	Storage Condition	Sampling Frequency
**Open-14_Light/RT_**	Daylight RT	Every 14 days
**Open-45_Light/RT_**	Daylight RT	Every 45 days
**Open-14_Dark/RT_**	Dark RT	Every 14 days
**Open-45_Dark/RT_**	Dark RT	Every 45 days
**Open-14_Cold_**	Cold (4 °C)	Every 14 days
**Open-45_Cold_**	Cold (4 °C)	Every 45 days

RT: Room Temperature.
